# Intact vagal gut‐brain signalling prevents hyperphagia and excessive weight gain in response to high‐fat high‐sugar diet

**DOI:** 10.1111/apha.13530

**Published:** 2020-07-22

**Authors:** Molly McDougle, Danielle Quinn, Charlene Diepenbroek, Arashdeep Singh, Claire de la Serre, Guillaume de Lartigue

**Affiliations:** ^1^ Department of Pharmacodynamics University of Florida Gainesville FL USA; ^2^ Center for Integrative Cardiovascular and Metabolic Disease University of Florida Gainesville FL USA; ^3^ The John B. Pierce Laboratory New Haven CT USA; ^4^ Department of Cellular and Molecular Physiology Yale Medical School New Haven CT USA; ^5^ Department of Foods and Nutrition University of Georgia Athens GA USA

**Keywords:** fat, obesity, palatability, post‐ingestive, sugar, vagus nerve

## Abstract

**Aim:**

The tools that have been used to assess the function of the vagus nerve lack specificity. This could explain discrepancies about the role of vagal gut‐brain signalling in long‐term control of energy balance. Here we use a validated approach to selectively ablate sensory vagal neurones that innervate the gut to determine the role of vagal gut‐brain signalling in the control of food intake, energy expenditure and glucose homoeostasis in response to different diets.

**Methods:**

Rat nodose ganglia were injected bilaterally with either the neurotoxin saporin conjugated to the gastrointestinal hormone cholecystokinin (CCK), or unconjugated saporin as a control. Food intake, body weight, glucose tolerance and energy expenditure were measured in both groups in response to chow or high‐fat high‐sugar (HFHS) diet. Willingness to work for fat or sugar was assessed by progressive ratio for orally administered solutions, while post‐ingestive feedback was tested by measuring food intake after an isocaloric lipid or sucrose pre‐load.

**Results:**

Vagal deafferentation of the gut increases meal number in lean chow‐fed rats. Switching to a HFHS diet exacerbates overeating and body weight gain. The breakpoint for sugar or fat solution did not differ between groups, suggesting that increased palatability may not drive HFHS‐induced hyperphagia. Instead, decreased satiation in response to intra‐gastric infusion of fat, but not sugar, promotes hyperphagia in CCK‐Saporin‐treated rats fed with HFHS diet.

**Conclusions:**

We conclude that intact sensory vagal neurones prevent hyperphagia and exacerbation of weight gain in response to a HFHS diet by promoting lipid‐mediated satiation.

**Abbreviations**
CCK: cholecystokinin
HFHS: high‐fat high‐sugar
NG: nodose ganglia
NTS: nucleus tractus solitarius
SAP: saporin

## 1 INTRODUCTION

The vagus nerve is the longest of the 12 cranial nerves. It broadly innervates many peripheral organs, underscoring its critical role in several physiological processes necessary for survival.^1^ Vagal efferent neurones located in the hindbrain send motor information from the brain to peripheral organs.^2^ Vagal sensory neurones, located in the nodose ganglia (NG), convey interoceptive feedback from each organ to the brain.^3^ Thus, vagal neurones located in anatomically distinct sites provide bidirectional information between peripheral organs and the brain. In both mice and rats, sensory vagal fibres extensively outnumber motor fibres in transverse sections of the vagus nerve,^4^, ^5^ suggesting a primary role of the vagus nerve in providing continuous homoeostatic feedback to the brain. At the level of NG, vagal sensory neurones can be categorized by organ innervation at least in mice,^3^ but no discernable anatomical organization for these ensembles has been identified. Within organs, the structural morphology of sensory vagal terminals distinguishes mechanosensitive and chemosensitive neurones,^6^ suggesting that vagal afferent neurones are involved in sensing multiple physiological events in peripheral organs.

The gastrointestinal tract is particularly densely innervated by the vagus nerve.^6^ In addition to its role in digestion and absorption, there is substantial evidence indicating that the vagus nerve controls food intake control in rodents and humans.^7^, ^8^, ^9^, ^10^, ^11^ Receptors capable of sensing fats,^12^, ^13^ carbohydrates,^14^ gut hormones^3^, ^15^, ^16^, ^17^, ^18^ and stretch^19^ are all expressed by NG neurones. Furthermore, each of these stimuli increases firing activity in whole vagus nerve electrophysiological recordings.^3^, ^20^, ^21^, ^22^, ^23^, ^24^, ^25^, ^26^, ^27^, ^28^, ^29^, ^30^ Post‐prandial signalling is blunted in response to chemical or physical lesioning, as demonstrated by reduced neuronal activity in the nucleus tractus solitarius (NTS), the central site of vagal sensory termination.^31^ Individual stimuli such as lipids,^32^ carbohydrates^33^ and gastric distension^34^ also require an intact vagus nerve to activate NTS neurones. Failure to activate vagal afferent neurones, by removal of the ingesta during a meal, results in continuous eating in rats,^35^ underlying the crucial role of post‐ingestive gut‐brain signalling in meal termination. Furthermore, optogenetic and chemogenetic stimulation of gastric and duodenal innervating vagal neurone populations acutely reduce food intake in mice.^7^, ^36^ Thus, the vagus nerve is anatomically positioned within the gut and is necessary and sufficient to convey moment‐to‐moment feedback to the brain about the quantity and makeup of a meal to trigger satiation.

There is evidence that repeated inhibition of satiation at each meal by disrupting vagal gut‐brain signalling results in long‐term metabolic consequences. Eating palatable calorie‐dense diets rich in fat and/or sugar for a prolonged period reduces the sensitivity of vagal afferent neurones to tension,^37^, ^38^ satiation hormones (eg Cholecystokinin [CCK])^37^, ^39^, ^40^, ^41^, ^42^, ^43^, ^44^ and intestinal nutrients^12^, ^45^, ^46^, ^47^ in both mice and rats. In rats, reduced vagal sensing coincides with the onset of hyperphagia and is associated with increased body weight.^48^ In a genetic mouse study, inhibition of vagal signalling resulted in a reduction of vagal afferent sensitivity to CCK and was sufficient to induce hyperphagia and weight gain.^49^, ^50^ Conversely, chronic vagal stimulation reduces food intake and body weight in many different animal species,^51^, ^52^, ^53^, ^54^, ^55^, ^56^ including humans.^57^ These data support a role for the vagus nerve in long‐term control of food intake by reducing postprandial feedback from the gut to the brain at every meal that could promote overeating in obesity. Based on this hypothesis it would be anticipated that inhibition of the vagus nerve would cause hyperphagia and increased body weight. In contradiction, previous studies in which vagal signalling is inhibited using surgical,^58^, ^59^ chemical^60^, ^61^ or genetic^62^, ^63^, ^64^, ^65^ approaches have little effect on daily cumulative chow intake. We hypothesize that the lack of specificity of the lesioning approaches to target sensory information for the gut and aggregation of studies using different diets has led to the erroneous conclusion that the vagus nerve only has a short‐term role in the control of food intake.

The majority of what is known about the function of the sensory vagal neurones relies on its anatomy, or techniques that lack precision and have several pitfalls. Vagus nerve stimulation is primarily applied unilaterally to the cervical vagus nerve which non‐selectively activates both sensory and motor arms of the vagus nerve with little consensus regarding stimulation parameters. Furthermore, chronic cervical vagal stimulation cause elevated fasting blood glucose levels and impairs glucose tolerance in rats.^66^ Inhibition achieved by vagotomy abolishes both the sensory and motor arms of the vagus. Capsaicin is not necessarily specific to the vagus nerve and only abolishes a subpopulation of unmyelinated sensory neurones and partially ablates motor neurones.^67^ Subdiaphragmatic vagal deafferentation approach improves on vagotomy and capsaicin by leaving 50% of motor control intact, but similarly lacks organ specificity.^68^ While more targeted viral‐mediated RNA interference approaches have been employed,^50^, ^69^, ^70^ this requires a priori knowledge of the signalling receptor to be knocked down. To address these issues, we have recently validated a novel pharmacotoxin approach, in which the neurotoxin saporin (SAP) is conjugated to the gastrointestinal hormone CCK (CCK‐SAP), for selectively deleting CCK receptor‐expressing sensory vagal neurones that innervate the stomach and small intestine.^71^ This approach ablates approximately 80% of sensory neurones innervating the proximal gut, while sparing motor neurones of the vagus nerve.^71^ It is important to note that ablating CCK‐A receptor‐expressing neurones with this approach, will delete the majority of vagal‐gut‐brain signalling rather than exclusively impairing CCK signalling. Because CCK receptor‐expressing neurones are involved in multimodal response including mechanosensation, anorectic signalling and orexigenic signalling,^36^ CCK‐SAP injections in the NG causes vagal deafferentation rather than just CCK‐receptor blockade. We utilize this novel gut‐specific vagal deafferentation approach to evaluate the role of gut‐brain signalling in the control of energy balance in rats fed either chow or high‐fat high‐sugar (HFHS) diets. We hypothesized that selective vagal deafferentation of the gut would differentially impair energy balance, glucose tolerance and gut nutrient sensing.

## 2 RESULTS

### 2.1 Vagal deafferentation increases number of meals in chow‐fed rats

**FIGURE 1:**
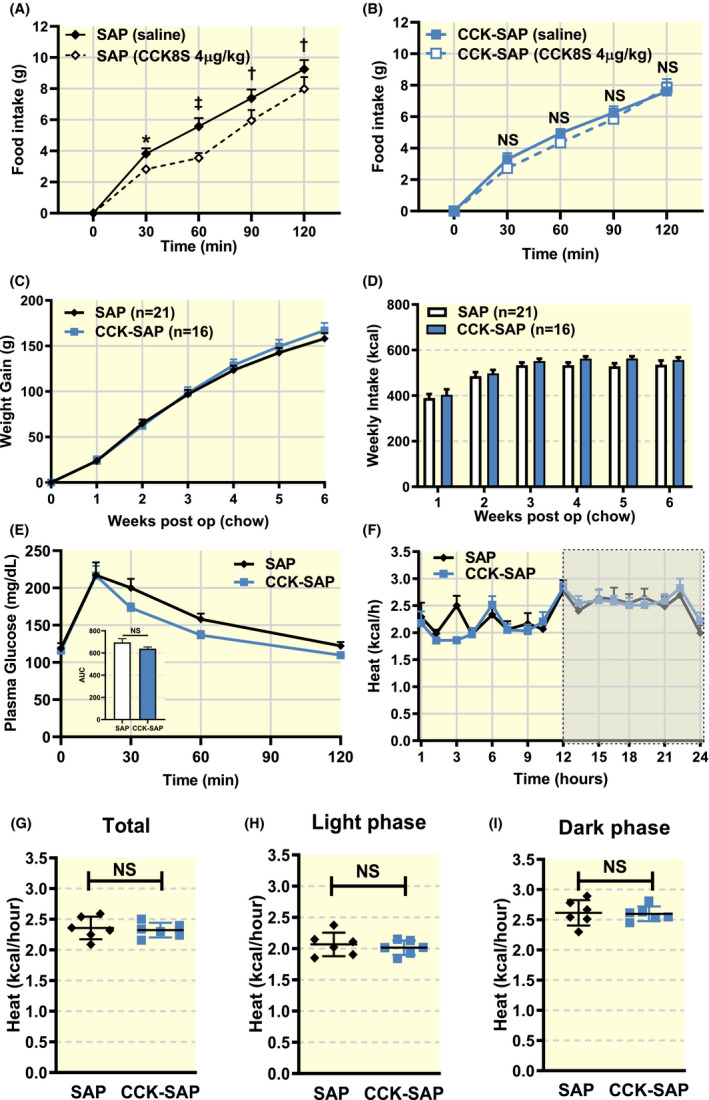
Gut‐specific vagal deafferentation has no long‐term effect on metabolism in lean, chow‐fed rats. Rats received bilateral NG injection of SAP (400 ng/side, n = 21) or CCK‐SAP (400 ng/side, n = 16). A‐B, CCK8S (IP 4 µg/kg) reduces food intake at 30‐120 minutes in (A) SAP injected( (n = 9, two‐way ANOVA, interaction F(4,32) = 3.662, *P* = .0145, Holm‐Sidak post hoc analysis, *P < .05, ^†^ *P* < .01, ^‡^ *P* < .0001) but not (B) CCK‐SAP injected rats (n = 7, two‐way ANOVA, interaction F(4,24) = 1.904, *P* = .1424, NS). C, Post‐op,CCK‐SAP had no effect on body weight gain (n = 21/16, two‐way ANOVA, interaction F(6,210) = 1.0, *P* = .4263; Holm‐Sidak post hoc analysis NS). D, In chow‐fed conditions, there was no difference in weekly caloric intake in either group. (n = 21/16, two‐way ANOVA, interaction F(5,175) = 0.4263, *P* = .83; Holm‐Sidak post hoc analysis NS). E, There is no difference in glucose tolerance between groups (n = 6, two‐way ANOVA, interaction F(4,52) = 0.9778, *P* = .4277; Holm‐Sidak post hoc analysis NS). Insert shows no difference in AUC between groups (two‐tailed unpaired *t*‐test, *P* = .1289). F‐I, Energy expenditure was measured in a metabolic chamber. F, Energy expenditure (kcal/hour) is not significantly different between SAP or CCK‐SAP (treatment (n = 6, two‐way ANOVA, interaction F(16,160) = 1.106, *P* = .3540; Holm‐Sidak post hoc analysis NS). G, Average kcal expended per hour during 24th day (n = 6, two‐tailed unpaired *t*‐test, *P* = .7057), H light phase (n = 6, two‐tailed unpaired *t*‐test, *P* = .5673), and I dark phase (n = 6, two‐tailed unpaired *t*‐test, *P* = .8642) were not affected by vagal deafferentation

To determine the role of vagal sensory neurones in controlling energy homoeostasis, we generated two groups of animals either lacking vagal gut‐brain signalling followed by bilateral NG injection of CCK‐SAP (400 ng/side, n = 16) or with normal vagal signalling that received bilateral NG injection of the control SAP (400 ng/side, n = 21). Pre‐surgical body weight was not significantly different (SAP: 277 ± 3.8 g, CCK‐SAP: 275 ± 6.6 g). We verified CCK‐SAP induced lesioning by performing a CCK‐induced satiation test 6 weeks after the surgery. CCK8S (4 μg/kg; IP) reduced food intake at 30‐120 minutes in SAP‐treated rats (Figure 1A), but not in CCK‐SAP‐treated rats (Figure 1B). Over the 6 weeks post‐op during which rats were maintained on the chow diet, we observed no differences in body weight gain between groups (Figure 1C). CCK‐SAP had no effect on weekly chow intake over the 6 weeks after surgery compared to SAP controls (Figure 1D). We performed an IP glucose tolerance test (IPGTT, Figure 1E) 5 weeks after surgery and found that vagal deafferentation of the gut did not affect glucose tolerance. In a separate cohort of rats, we found that 5 weeks after surgery, the average energy expenditure over 3 days was not different between chow‐fed SAP and CCK‐SAP‐treated rats (Figure 1F,G) irrespective of whether the animals were in the light (Figure 1H) or dark (Figure 1I) phase.

**FIGURE 2:**
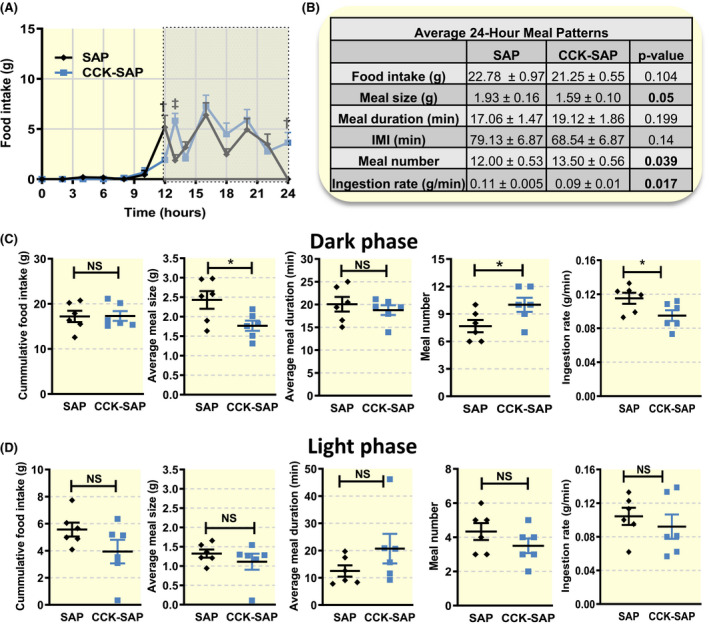
Gut‐specific vagal deafferentation alters meal patterns in lean chow‐fed rats. Food intake patterns were measured in a subset of rats receiving bilateral NG injection of SAP (400 ng/side, n = 6) or CCK‐SAP (400 ng/side, n = 6). A, Vagal deafferentation shifted the anticipatory circadian cycle evoked changes in food intake (n = 6, two‐way ANOVA, interaction F(13,182) = 3.823, *P* < .0001, Holm‐Sidak post hoc analysis, ^†^ *P* ≤ .01, ^‡^ *P* < .001). B‐D, Meal patterns differed between groups. B, Over the course of the full day and (C) in the dark phase CCK‐SAP‐treated rats ate slower and smaller meals with no net change in daily food intake due to greater number of meals (two‐tailed unpaired *t*‐test, *P < .05). D, In the light phase there were no differences in meal patterns between groups (two‐tailed unpaired *t*‐test, NS)

Based on previous work highlighting a role for vagal signalling in the short‐term control of food intake, we monitored continuous ad libitum chow intake using a BioDAQ system. There was a shift in the time of day that food was consumed between groups; SAP‐treated rats started eating before dark onset and stopped before light onset, while CCK‐SAP‐treated rats started eating once the dark phase had started and continued eating until the end of the dark phase (Figure 2A). In addition, meal patterns were significantly altered in CCK‐SAP rats; meal number was increased compared to control SAP rats and this was associated with a reduction in meal size and ingestion rate, suggesting reduced satiety (Figure 2B). These effects were observed in the dark phase (Figure 2C) but were absent in the light phase (Figure 2D).

### 2.2 Vagal deafferentation exacerbates weight gain following exposure to HFHS diet

**FIGURE 3:**
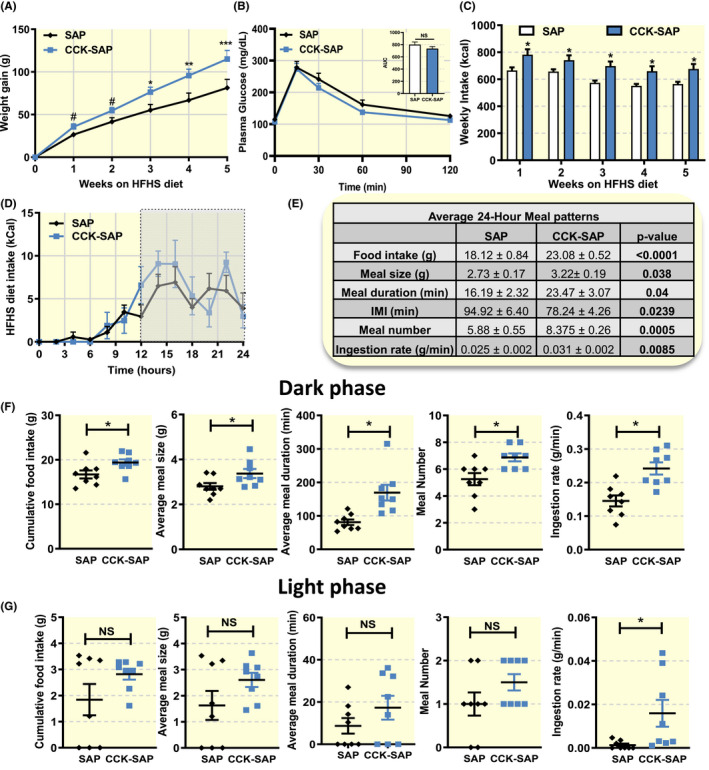
Vagal deafferentation causes weight gain and hyperphagia in rats fed high‐fat high‐sugar (HFHS) diet. 6 weeks post‐op, chow‐fed rats were switched to HFHS diet (A) CCK‐SAP rats gained more weight (n = 7/8, two‐way ANOVA, interaction F(5,60) = 5.636, *P* = .0003, Holm‐Sidak post hoc analysis, # *P* = .06 *P < .05, ^†^ *P* < .01, ^‡^ *P* < .001). B, There was no effect of dietary change on glucose tolerance between group (n = 7/8, two‐way ANOVA, interaction F(4,48) = 0.3731, *P* = .8267, Holm‐Sidak post hoc analysis, NS). Insert shows no difference in AUC (two tailed unpaired *t*‐test, *P* = .2406). C, In response to HFHS diet, CCK‐SAP‐treated rats ate more calories than SAP rats. (n = 7/8, two‐way ANOVA, interaction F(4,52) = 0.9384, *P* = .4491, Holm‐Sidak post hoc analysis, *P < .05). D‐G, Meal patterns of CCK‐SAP and SAP rats were recorded at 9 weeks post‐op averaging three consecutive days of food intake in rats acclimated to BioDAQ. D Both groups of animals consume food at the same time of day (n = 7/8, two‐way ANOVA, interaction F(12,144) = 1.012, *P* = .4410, Holm‐Sidak post hoc analysis, NS). E, Daily food intake was increased as a results of increased meal size, duration, ingestion rate and increased meal number (n = 8, two‐tailed unpaired *t*‐test). These effects were mediated altered meal patterns in (F) the dark phase, rather than (G) the light phase (n = 8, two‐tailed unpaired *t*‐test, *P < .05)

At 6 weeks post‐op, a subset of rats (n = 8/group) was switched from chow to HFHS diet. CCK‐SAP‐treated rats trended (*P* < .06) to gain more weight in the first 2 weeks of HFHS diet. Body weight gain was significantly elevated in CCK‐SAP rats from 3 weeks onwards compared to SAP controls (Figure 3A). Interestingly, despite significant weight gain differences between groups at 3 weeks, IPGTT was not different (Figure 3B). However, weekly food intake was significantly greater at each time point compared to controls (Figure 3C). The time of day at which food intake occurred was similar between groups (Figure 3D). The increased cumulative food intake resulted from increases in ingestion rate, meal size, meal duration and meal number (Figure 3E). The altered meal patterns were restricted primarily to the dark phase (Figure 3F), although the ingestion rate was also increased in the light phase (Figure 3F).

**FIGURE 4:**
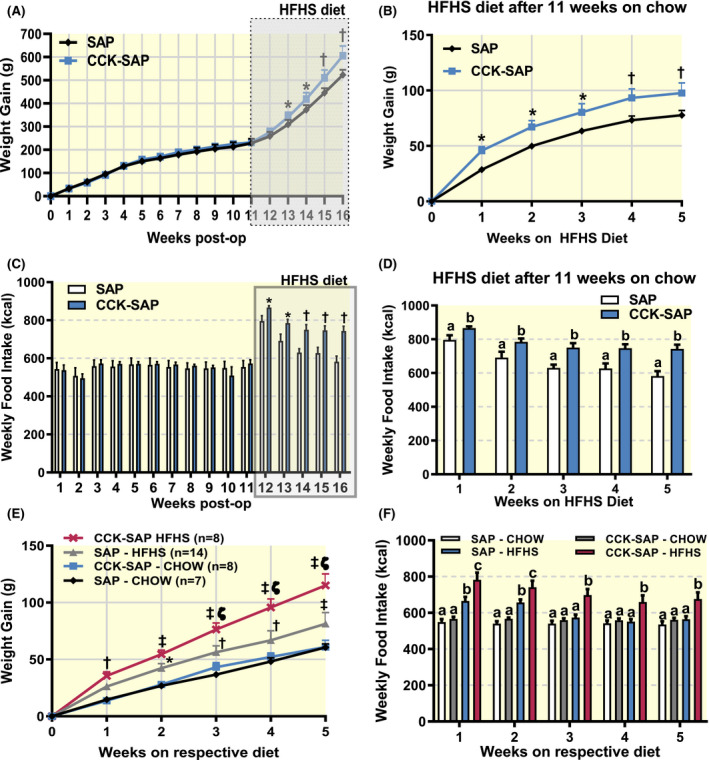
HFHS diet causes hyperphagia and weight gain in CCK‐SAP‐treated rats. Rats received bilateral NG injection of SAP (400 ng/side) or CCK‐SAP (400 ng/side) and maintained on chow for 11 weeks post‐op before switching to HFHS diet (n = 8/group). A, There was no post‐op body weight gain during 11 weeks on chow, but CCK‐SAP‐treated rats started gaining more weight upon transitioning to HFHS diet (n = 8, two‐way ANOVA, interaction F(16,112) = 2.585, *P* = .0019, Benjamini, Krieger and Yekutieli post hoc analysis with FDR 0.05, *P < .05, ^†^ *P* < .01). B, Normalizing weight gain to the body weight upon dietary switch, confirms greater weight gain in CCK‐SAP‐treated rats compared to SAP controls (n = 8, two‐way ANOVA, interaction F(5,60) = 3.381, *P* = .0093, Holm Sidak post hoc analysis, *P < .05, ^†^ *P* < .01). C, Weekly food intake remained similar between groups for the rats on chow for 11. Caloric intake increased in response to HFHS diet in both groups compared to chow (n = 8, two‐way ANOVA, interaction F(15, 210) = 4.457, *P* < .0001, Benjamini, Krieger and Yekutieli post hoc analysis, *P < .05, ^†^ *P* < .01). D, Caloric intake was greater in CCK‐SAP‐treated, compared to SAP‐treated, rats (n = 8, two‐way ANOVA, interaction F(4,52) = 2.176, *P* = .0845, Holm Sidak, different letters^a,b^ indicate *P* < .05). E‐F, Analysis of age‐matched 6 weeks post‐surgery of bilateral SAP or CCK‐SAP in NG on respective diets. E, HFHS diet increases body weight gain more rapidly in CCK‐SAP‐treated compared to SAP‐treated rats (n = 7‐14, two‐way ANOVA, interaction F(15,165) = 10.53, *P* < .001, Holm‐Sidak post hoc analysis, *P < .05, ^†^ *P* < .01, ^‡^ *P* < .001 compared to chow, and ^ζ^p < 0.01 compared to SAP‐HFHS group). F, HFHS‐fed CCK‐SAP‐treated rats consume more calories per week than all other groups at time point, while HFHS‐fed SAP‐treated rats normalize intake compared to chow‐fed rats in the last 3 weeks of the study (n = 7‐14, two‐way ANOVA, interaction F(15,165) = 10.53, *P* < .001, Holm‐Sidak post hoc analysis, different letters^a,b,c^ indicate *P* < .05 between groups)

To determine whether the increased body weight gain and food intake in the CCK‐SAP‐treated animal was the direct consequence of dietary change, rather than a coincidence related to the age of the animals or a delayed effect of the surgery, we repeated the experiment in a new cohort of rats. As before rats received a bilateral injection of CCK‐SAP (n = 8) or SAP (n = 8) at the same age, but this time were maintained on chow for 11 weeks post‐op before switching to HFHS diet for an additional 5 weeks. SAP‐ and CCK‐SAP‐treated rats weighed the same (Figure 4A) and consumed the same amount of food (Figure 4C) at every time point over 11 weeks while maintained on chow. Similar to the CCK‐SAP‐treated rats in Figure 3, the CCK‐SAP‐treated rats gained more weight once switched to HFHS diet (Figure 4B). We also found that the CCK‐SAP rats consumed more calories while on HFHS diet compared to SAP controls (Figure 4D).

We combined the weight gain and food intake data from the age‐matched rats fed HFHS diet (Figure 3) or chow (Figure 4) at 6‐11 weeks after NG surgery. Although both SAP and CCK‐SAP‐treated rats gained more weight when fed HFHS diet, it is clear that vagal deafferentation exacerbated weight gain in response to the diet (Figure 4E). Notably, the CCK‐SAP‐treated animals weighed more than lean animals from week 1 while SAP‐treated animals started to weigh more than lean controls from week 2, and HFHS‐fed CCK‐SAP rats weighed more that HFHS‐fed SAP rats (Figure 4E). In addition, CCK‐SAP animals consumed more calories at each time point when fed HFHS compared to CCK‐SAP rats fed chow (Figure 4F); while SAP animals consumed more calories in the first couple of weeks before reducing their food consumption to match the caloric intake of chow‐fed rats after 3 weeks on HFHS diet (Figure 4F).

### 2.3 Vagal deafferentation blocks post‐ingestive lipid‐mediated meal termination

**FIGURE 5:**
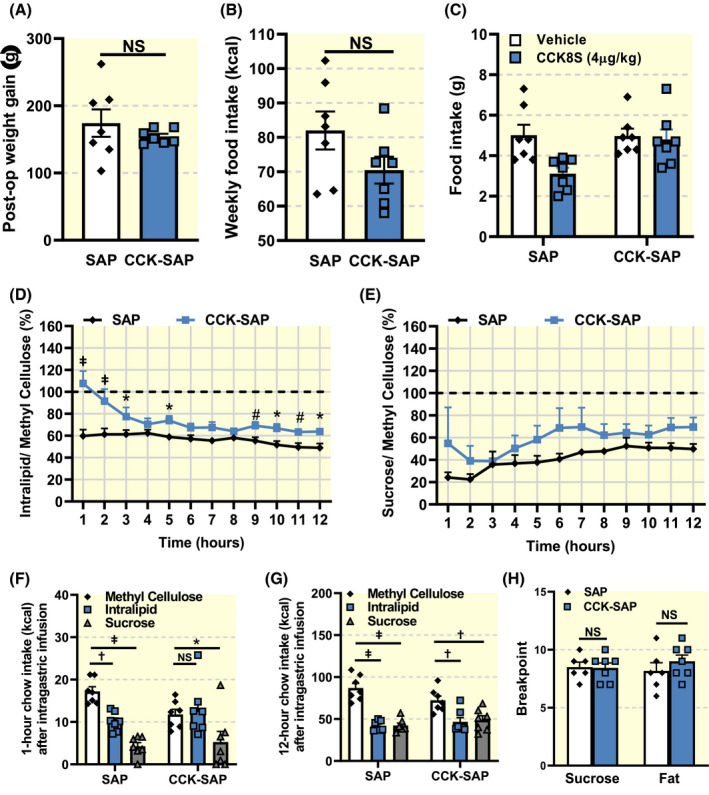
Vagal deafferentation causes inhibition of lipid‐mediated satiation. A, Weight gain was similar between groups (n = 7, two tailed unpaired *t*‐test, *P* = .3612). B, Chow intake was not significantly different between groups (n = 7, two tailed unpaired *t*‐test, *P* = .1149). C, 1 hour food intake in response to i.p. CCK (4 µg/kg) was reduced in SAP, but not in CCK‐SAP‐treated rats (n = 7, two way RM ANOVA, Interaction F(1,12) = 6.392, *P* = .0265, Holm‐Sidak post hoc analysis ^†^ *P* < .01). D, SAP but not CCK‐SAP animals reduce food intake following intra‐gastric pre‐load of lipids (n = 7, two way ANOVA Interaction F(11,132) = 4.003, *P* < .0001, Benjamini and Hochberg post hoc analysis with FDR of 0.05 ^#^ *P* < .06, *P < .05, ^‡^ *P* < .001). E, Both SAP and CCK‐SAP animals reduce food intake following intra‐gastric pre‐load of sucrose (n = 7, two way ANOVA Interaction F(11,132) = 0.6730, *P* = .7619, Benjamini and Hochberg post hoc analysis with FDR of 0.05, NS). F, Caloric intake 1 hour after intra‐gastric pre‐load of methyl cellulose, fat or sugar (n = 7, two way ANOVA, Interaction F(2,24) = 3.878, *P* = .0347, Dunnett's post hoc analysis, *P < .05, ^†^ *P* < .01, ^‡^ *P* < .001). G, Caloric intake 12 hours after intra‐gastric pre‐load of methyl cellulose, fat or sugar (n = 7, two way ANOVA, Interaction F(2,24) = 4.944, *P* = .0159, Dunnett's post hoc analysis, ^†^ *P* < .01, ^‡^ *P* < .001). H Breakpoint in the progressive ratio licking test was unchanged across groups irrespective of macronutrient reward (n = 7, Two‐way RM ANOVA, Interaction F(1,8) = 0.8037, *P* < .05, Holm‐Sidak post hoc analysis, NS)

We hypothesized that the increased intake of HFHS diet in CCK‐SAP‐treated rats was either a consequence of reduced post‐ingestive feedback from the gut, and/or increased wanting for the highly palatable diet. Both these putative mechanisms were tested in lean chow‐fed rats exposed to equicaloric fat or sugar. Importantly, the rats were tested while fed chow diet to prevent the well‐characterized confounds from reduction in vagal sensory neurone sensitivity to nutrients,^12^, ^45^, ^46^, ^47^ hormones^37^, ^39^, ^40^, ^41^, ^42^, ^43^, ^44^ and tension^37^, ^38^ in animals chronically fed HFHS diet. The rationale for opting to study the mechanism of CCK‐SAP‐induced hyperphagia in chow‐fed rats that are not hyperphagic is supported by the fact that CCK‐SAP rats started to overconsume immediately upon first exposure to HFHS diet (Figures 3C and 4D,F). Confirming our previous results in Figures 1 and 4, we observed no group differences in body weight gain (Figure 5A) or average food intake (Figure 5B) in this cohort of chow‐fed animals over the 13 weeks of the study (n = 7/group). We verified vagal deafferentation of the gut in this group of rats using a CCK (IP, 4 µg/kg) satiation test. SAP animals significantly decrease 1 hour food intake in response to exogenous CCK‐8, while CCK‐SAP animals exhibited no change in food intake after CCK administration (Figure 5C).

To determine the role of post‐ingestive feedback in HFHS diet‐induced hyperphagia, chow‐fed SAP or CCK‐SAP‐treated rats were infused 10 mL of either 37.5% fat, 75% sucrose or 2% methyl cellulose into the stomach at a rate of 1 mL/min. To account for the individual and group differences in meal patterns, we designed the experiments to be a within‐animal design in which each animal acted as its own control. Furthermore, we controlled for distension effects by using methyl cellulose as a baseline. A 2% methyl cellulose pre‐load was used as a control as it is composed of non‐metabolizable nutrients and has a similar viscosity to fat and sugar solutions. A saline pre‐load was also performed, and similar results were observed (data not shown). Importantly, chow intake in response to a pre‐load of 2% methyl cellulose was similar between SAP‐ and CCK‐SAP‐treated animals (Figure 5E,G). In response to an intra‐gastric pre‐load of a fat solution, SAP‐treated rats markedly decreased their food intake for 12 hours, when compared to a methyl cellulose pre‐load (Figure 5D,F,G). By contrast, CCK‐SAP‐treated animals did not reduce intake in the first 2 hours following the fat pre‐load compared to a methyl cellulose pre‐load, and maintained significantly higher food intake than SAP controls at the majority time points over 12 hours (Figure 5D,F,G). Furthermore, both CCK‐SAP and SAP rats significantly decreased food intake following an intra‐gastric pre‐load of an equicaloric sucrose solution (Figure 5E‐G). Together, these data suggest that CCK‐SAP animals have dampened lipid‐mediated, but not sugar‐mediated, reduction in food intake.

To determine whether CCK‐SAP‐treated rats are more motivated to consume palatable HFHS diet, we tested the willingness of the animals to work for the taste of fat or sugar. The same lean chow‐fed SAP or CCK‐SAP‐treated rats were trained to lick for equicaloric solutions of 10% fat or 20% sucrose before performing a progressive ratio licking task,^72^ in which exponentially increasing numbers of licks were required for either fat or sugar delivery. The breakpoint, the point at which the animals ceased licking the dry sipper to get the reward, presented as the last reward achieved, was used to determine the willingness of the animals to work for fat or sugar. Similar breakpoints were recorded for both groups of rats licking for either fat or sugar reward (Figure 5H), suggesting that gut deafferentation failed to increase the willingness to work for oral intake of either fat or sugar.

## 3 DISCUSSION

The study aimed to determine the role of sensory vagal neurones that innervate the gut in the control of energy balance. On utilizing a novel highly selective approach for vagal deafferentation of the gut, we found that rats lacking gut‐brain signalling did not affect energy expenditure or glucose homoeostasis, but significantly altered meal patterns in chow‐fed conditions. When the same animals were switched to a HFHS diet, cumulative food intake was markedly increased over multiple weeks resulting in increased body weight. These data suggest a role for the gut‐brain axis in both short‐term and long‐term control of food intake depending on nutrient availability. By directly infusing nutrients into the gut we demonstrate that vagal deafferentation impairs fat, but not sugar satiation, suggesting that impaired post‐ingestive feedback of fat is sufficient to promote hyperphagia and weight gain in response to a HFHS diet.

CCK receptors are G‐protein coupled receptors that are expressed in NG neurones.^15^, ^73^ Upon binding of CCK‐SAP to CCK receptors, the internalization of the ligand‐receptor complex results in the saporin‐mediated ablation of the cell. Thus this approach not only abolishes CCK signalling but actually deletes these neurones and therefore prevents all signalling by NG cells that express CCK receptors. Using a retrograde tracing approach, we previously reported that the population of vagal sensory neurones that are ablated in response to CCK‐SAP injection primarily innervate the mucosal and muscular layers of the stomach and at least the upper part of the small intestine.^71^ Previous work supports a role for CCK sensitive vagal neurones in both gastric and duodenal mechanoreceptive fibre firing^74^ and that CCK‐sensitive neurones are required for mediating the satiating effect of nutrients.^75^ CCK receptor‐expressing vagal sensory neurones co‐express hormone receptors associated with anorectic,^18^, ^76^ as well orexigenic function,^77^, ^78^, ^79^ suggesting that these neurones can convey conflicting sensory signals. Furthermore, a recent RNA profiling study in mice confirm that CCK receptor‐expressing NG neurones innervate the length of the gastrointestinal tract and that these neurones co‐express (a) Glucagon‐like peptide‐1 receptor (GLP1R), a marker of gastric mechanosensitive neurones, (b) Oxytocin receptor (OxtR), a marker of intestinal mechanosensitive neurones as well as (c) vasoactive intestinal peptide (VIP) a marker of intestinal chemosensitive neurones.^36^ In line with this, we previously demonstrated that a dose of exogenous GLP1 that causes satiation in SAP control rats fails to inhibit food intake in CCK‐SAP‐treated rats.^71^ Importantly, Bai et al found that stimulation of subpopulations of CCKR neurones in the NG, specifically those that co‐express with GLP1R or OxtR, decreased food intake.^36^ Thus, the ablation of CCK receptor‐expressing NG neurones prevents multimodal signalling from the gut to the brain, and is therefore a useful tool for vagal deafferentation of the gut.

CCK‐SAP ablated rats have altered meal patterns on a chow diet, characterized by increased meal number and frequency, and decreased meal size and ingestion rate. This may suggest that vagal gut‐brain circuits primarily control satiety, which prevents eating between meals, rather than satiation, which terminates a meal. Consistent with our results implicating a role of the vagus nerve in satiety rather than satiation, subdiaphragmatic vagotomy causes smaller, more frequent meals in both rats fed with liquid chow diet^80^ and mice fed chow.^81^ In addition, i.p. capsaicin‐induced deafferentation in chow‐fed rats increased meal frequency.^82^ Thus, irrespective of the mechanism by which vagal signalling is disrupted, the consistent finding in each of these studies is that the total number of meals is increased in chow‐fed animals and this is usually accompanied by a reduced meal size. The current work significantly improves on the lack of organ specificity following vagotomy and capsaicin treatment, and provides direct evidence that gut‐specific sensory signalling is necessary for satiety. Notably, these results conflict with the CCK signalling literature. CCK administration decreases meal size followed by a compensatory increase in meal number,^83^ while inhibition of CCK receptor by genetic^84^, ^85^, ^86^ or pharmacological^87^, ^88^ methods increases meal size and decreases meal number. Our data suggest that gut‐brain vagal sensory signalling cannot be defined as an aggregate of individual signalling components.

Interestingly, when these same rats were given access to a palatable energy‐dense diet high in fat and sugar, vagal deafferentation of the gut increased cumulative food intake. Similar to that observed in lean rats, CCK‐SAP increased the number of meals; however, it also increased meal size, duration and ingestion rate, resulting in hyperphagia compared to SAP‐treated HFHS‐fed controls. The meal patterns changes in rats were restricted to the dark phase irrespective of diet, presumably because rodents consume the majority of their food in the dark. Interestingly, other feeding studies in which vagal sensory signalling was impaired also reported disruption of meal patterns only in the dark,^49^, ^50^, ^70^ and is therefore a conserved phenomenon. In HFHS‐fed animals, vagal gut‐brain signalling conveys signals that affect both satiation and satiety, resulting in pronounced hyperphagia. This result was very robust, occurring in all rats with confirmed desensitization to CCK‐induced satiation in multiple batches of rats irrespective of when the HFHS diet was introduced post‐op, suggesting that CCK‐SAP‐induced hyperphagia was a result of the diet rather than age or time after surgery. Similar increases in meal size have been reported in capsaicin‐treated animals fed novel high‐fat foods in the first few days post‐surgery compared to controls.^9^, ^10^, ^11^ Similarly, subdiaphragmatic vagal deafferentation causes an increase in meal size in rats fed a calorically dense liquid diet,^58^ although this was accompanied by a decreased meal frequency. The difference between these previous studies and our current findings are likely caused by retaining intact vagal motor function. In mice lacking Nav 1.8 neurones, which include a subpopulation of vagal afferent neurones,^89^ there was no effect on meal patterns in mice fed with HFHS diet compared to littermate controls.^90^ It is likely that these mice develop compensatory mechanisms from lacking Nav1.8 neurones from birth, or that there is sufficient vagal innervation of the gut remaining to communicate nutrient information to the brain, especially since fat‐induced cFos signalling in the NTS was not different between ablated and control mice.^90^ We find that highly specific vagal deafferentation of the gut in adult rats increases food intake and impairs fat sensing, suggesting that a fully functional vagus prevents overeating and excessive weight gain when challenged with a high‐calorie density diet.

The potential protective role of the intact vagus nerve in preventing exacerbation of weight gain in response to high‐calorie diets may be relevant in the progression of obesity. Chronic exposure to HFHS diet results in reduced sensitivity of NG neurones to tension,^37^, ^38^ satiation hormones (eg, CCK),^37^, ^39^, ^40^, ^41^, ^42^, ^43^, ^44^ and intestinal nutrients.^12^, ^45^, ^46^, ^47^ Furthermore, post‐prandial neuronal activation in the NTS is significantly lower in obese, compared to lean, rats,^47^, ^91^ suggesting that gut‐brain signalling is severely blunted. Our previous work demonstrates that disrupted vagal signalling coincides with the onset of hyperphagia.^44^, ^50^ The exact mechanism by which vagal signalling is reduced in obesity remains unclear but may involve blunted receptor expression,^44^, ^92^, ^93^, ^94^, ^95^ impaired neuropeptide release^50^ and/or altered biophysical properties.^37^ Irrespective of mechanism it is clear that in obesity, loss of vagal signalling reduces gut‐brain signalling, and our new data demonstrate that this is sufficient for long‐term dysregulation of energy homoeostasis.

To determine the mechanism by which vagal deafferentation increased food intake and body weight, we tested the relative importance of post‐ingestive nutrient sensing as a feedback mechanism for meal termination. Intra‐gastric administration of fat pre‐load significantly inhibited food intake in SAP‐treated rats but failed to acutely reduce food intake in CCK‐SAP rats lacking gut‐brain signalling. Baseline food intake was slightly, but not significantly, lower following methyl cellulose pre‐load in the CCK‐SAP compared to control rats; however, it is unlikely that this accounts for the blunted response to fat for three reasons. Firstly, the study is a within‐animal design which accounts for baseline intake of individual animals, and controls for the group differences in meal patterns we report in Figure 2. Secondly, we observed the same blunted response to fat when normalized to saline. Finally, sugar pre‐loads continue to be reduced compared to methyl cellulose (or saline), suggesting that fat signalling was selectively inhibited. Together these data suggest that (a) vagal gut‐brain signalling is critical for fat sensing and monitoring, and (b) impaired fat‐specific post‐ingestive feedback is sufficient to trigger hyperphagia at least acutely. Certainly, the biased loss of fat sensing in response to targeted ablation of CCK receptor‐expressing sensory vagal neurones is consistent with previous data showing that CCK is released preferentially in response to fat and amino acids compared to carbohydrates.^96^, ^97^ There is extensive evidence that infusion of fats into the gut results in both meal termination^98^, ^99^ and appetition.^100^, ^101^ Previous work demonstrates that CCK‐SAP prevents intra‐gastric fat‐induced dopamine release, the ability of the animals to form a flavour conditioned preference for fat, or learn to self administer intra‐gastric fat.^7^ CCK, chemogenetic or optogenetic stimulation of this gut‐innervating vagal pathway causes a reduction of food intake, and many hallmark reward behaviours including conditioned flavour learning, conditioned place preference and self‐stimulation.^7^ Thus, in mice, both CCK and fat reduce food intake via a reward circuit that requires CCK receptor‐expressing vagal sensory neurones. In rats, nutrient‐induced satiation signals influence motivation to work for food.^102^ These data suggest an overlap in post‐ingestive fat mediated reward and meal termination through a vagal gut‐brain circuit. Based on these data we hypothesize that reduced post‐ingestive signalling of fat is sufficient to prevent appropriate meal termination and that chronic overeating at every meal promotes weight gain.

Intra‐gastric pre‐load of sucrose reduced subsequent chow intake similarly in both CCK‐SAP‐treated rats and SAP controls. In support of these findings, subdiaphragmatic deafferentation failed to inhibit glucose intake after a small gastric pre‐load of sugar, while fat had no effect.^58^ Interestingly, we found that intra‐gastric sucrose pre‐load was a more potent inhibitor of food intake than lipid pre‐load in SAP‐treated animals. Similar observations were previously reported in humans, where carbohydrate supplementation was a more potent appetite suppressor than fat supplementation at breakfast.^103^ The fact that sugar pre‐load equally reduced food intake in both SAP and CCK‐SAP‐treated rats suggests that the CCK‐receptor expressing vagal sensory neurones are not necessary for the appetite suppressant effects of intra‐gastric sucrose. A rapid rise in circulating glucose may act through a vagally independent mechanism via the hindbrain or hypothalamus, or an insulin‐mediated mechanism that is released in response to glucose that acts though a population of vagal afferent neurones that express insulin receptors^104^ but not CCK receptor. Alternatively, the high osmolarity from the very concentrated sucrose solution infused in the gut may cause aversion. We observed no difference in the rats’ willingness to work for either orally administered fat or sugar. Since small volumes were administered in the progressive ratio task, we expect negligible vagal signalling from the gut and thus no post‐ingestive reward. Therefore we conclude from the data that increased preference for the taste of the HFHS diet was not driving motivation to overeat. Little work has been done to address the role of the vagus nerve in taste perception. One study indicates that after subdiaphragmatic vagotomy rats no longer considered oral palatability when deciding how much food to eat.^105^ Specifically, vagotomized rats ate the same amount of three test meals in which one was regular chow, one was chow sweetened with sodium cyclamate and one was chow adulterated with quinine hydrochloride.^105^ Altogether, this suggests that although the CCK‐SAP rats consumed more of the HFHS diet, the increased palatability of the diet is not likely to be an important factor in the hyperphagia. However, future work of directly testing the taste profiles of different fat and sugar concentrations is warranted following vagal deafferentation of the gut.

While some studies involving the gut‐vagus‐brain axis indicate an ability of gut nutrient sensing vagal afferents to control glucose homoeostasis^70^, ^106^ and modulate energy expenditure in rodents,^107^, ^108^, ^109^ the specific vagal branch(es) mediating these effects remain unclear. In our current study, despite changes in meal patterns, the ablation of CCK‐sensitive vagal afferent neurones in rats did not affect glucose tolerance or whole‐body energy expenditure under chow‐fed conditions. Although our IPGTT data suggest that gut‐brain vagal signalling is not necessary for glucose metabolism after 3 weeks of HFHS diet, it is important to note that previous studies have reported that carbohydrate‐induced incretin release is sufficient for insulin release via a vagally mediated mechanism.^70^ Therefore, monitoring incretin release and additional time points should be tested alongside when conducting hyperglycaemic clamp experiments to fully assess the role of gut‐brain signalling in diet‐induced glucose homoeostasis. Duodenal infusions of lipid emulsions at doses that reduce meal size have been reported to increase the temperature in brown adipose tissue in rats, indicative of increased thermogenesis.^107^ Notably, the effect of BAT activation was reported to be (a) decreased with chronic high‐fat diet consumption, (b) abrogated by cervical vagotomy^108^ or (c) blocked by local intestinal application of the anaesthetic tetracaine, as well as by (d) peripheral administration of the CCK1R devazepide.^107^ BAT thermogenesis accounts for a small fraction of total energy expenditure, it is therefore possible that by measuring whole‐body energy expenditure, we missed a small change in thermogenesis and/or that this was compensated by a reduction in activity or basal metabolic rate. Furthermore, we did not measure whole‐body energy expenditure in the animals fed HFHS diet, therefore, whether vagal deafferentation of the gut affects energy expenditure in response to diet‐induced obesity needs further investigation.

A variety of diets ranging in physical form (solid vs liquid) and fat composition have been used in the past to assess the role of vagal signalling in the control of food intake. The distinction in the outcomes of vagal disruption in response to these different diets has been unclear. This combined with the lack of specificity in targeting selective organs or sensory arm of the vagus nerve has caused discordance about the function of gut‐to‐brain signalling in food intake. Here we have used a method for rapid and long‐lasting ablation of a targeted subpopulation of vagal sensory neurones that broadly sense both mechanical and chemical signals along the length of the gastrointestinal tract. We find that CCK receptor‐expressing vagal sensory neurones are necessary to control short‐term satiety. Interestingly, this gut‐brain circuit is necessary to prevent excessive overconsumption of fat, and inhibiting this post‐ingestive feedback mechanism promotes overeating and weight. Thus, a fully functioning vagus nerve prevents against long‐term weight gain caused by an obesogenic diet. Altogether these data identify a putative mechanism by which targeting the vagus nerve can be a useful therapeutic strategy for obesity.

## 4 MATERIALS AND METHODS

### 4.1 Animals and Housing

Adult male Wistar rats (220‐250 g starting bodyweight, Harlan, San Diego, CA and Envigo, Tampa, FL) were individually housed at 22°C under a 12‐h light‐dark cycle with ad libitum access to water and either chow (3.1 kcal/g, Teklad 2018, Envigo, Sommerset, NJ) or HFHS diet (45% calories from fat; 4.7 kcal/g, Research Diets D12451, New Brunswink, NJ), unless stated otherwise. Animals were allowed for 1 week to acclimate before any experiments were started. All experiments were approved by the University of Florida, and John B. Pierce Laboratory Institutional Animal Care and Use Committees (IACUC).

### 4.2 Peptides and drugs

Saporin (SAP) and CCK‐Saporin were obtained from Advanced Targeting Systems (San Diego, Ca). CCK‐8 was obtained from Bachem BioScience Inc (King of Prussia, PA).

### 4.3 Surgery

#### 4.3.1 Nodose ganglia injection

Before surgery, rats were fasted overnight with ad libitum access to water. Twenty minutes before surgery, rats received a subcutaneous injection of atropine sulphate (0.05 mg/kg; Henry Schein, Wallingford, CT) and carprofen (5.0 mg/kg; Henry Schein). Rats were anaesthetized with isoflurane (2% Isoflurane; Henry Schein). The rat was shaved from the chin to thorax and placed supine on a heated pad with a nose cone ventilator (SomnoSuite; Kent Scientific, Torrington, CT). A midline incision was made with a scalpel along the length of the neck; salivary glands and lymph nodes were retracted away from the midline. The sternohyoid and omohyoid were separated and retracted to expose the carotid artery and vagus nerve. The vagus nerve was separated from surrounding fascia and the carotid artery with fine tip forceps and retractors until the NG became accessible. A glass capillary (30 μm tip, beveled 30 degree angle) attached to a micromanipulator was used to position and puncture the NG and 1.5 µL volume of CCK‐SAP (250 ng/µL) or SAP (250 ng/µL) was injected with a Picospritzer III injector (Parker Hannifin, North Haven, CT) in the centre of the NG. The same procedure was repeated contralaterally before the skin was closed with sterile suture. Rats were allowed to recover under infrared heat until they chose to reside in the unheated side of the cage, at which point they were returned to their home cage, deprived of water for 6 hours and food overnight. We found that the previously used post‐op feeding regimen^71^, ^110^ did not improve recovery or survival, therefore post‐op rats received carprofen (5.0 mg/kg; SQ) and were returned to ad libitum access to chow from day 1.

#### 4.3.2 Gastric catheter implant

Rats were fasted overnight and received carprofen (5 mg/kg; SQ) 20 minutes prior to surgery. The back of the neck and the abdomen were shaved around the midline and rat was placed supine on a heated pad with a nose cone ventilator (SomnoSuite; Kent Scientific, Torrington, CT). A 15‐mm midline incision was made with a scalpel in the skin of the abdomen beginning at the sternum, followed by a similar incision in the linea alba of the muscle layer. The stomach was externalized and a 5‐mm purse suture was placed in the greater curvature near the fundus. Fine tip forceps were used to form a puncture in the centre of the purse suture and a catheter made from silicone tubing (SIL 047; Braintree Scientific, Braintree, MA) was inserted into the stomach. The purse suture was then tightened around the catheter and tied off to secure it in place. The stomach was returned to the abdominal cavity and a second 5‐mm purse suture was placed in the muscle layer of the abdomen 1 cm lateral to the midline incision, punctured with fine tip forceps and the open end of the catheter pulled through and secured by tying off the suture. The abdominal muscle layer was then closed with suture and the animal turned on its side. A small incision was made at the base of the skull and haemostats were used to blunt dissect the skin away from the muscle layer between the incision at the back of the neck and the abdominal incision, behind the shoulder, forming a tract for the catheter. The open end of the catheter was then pulled through to the back of the neck and secured using a purse suture around the opening. The skin of the abdomen was closed with suture and the cathether flushed with sterile saline prior to being capped. Rats were allowed to recover under infrared heat until they chose to reside in the unheated side of the cage, at which point they were returned to their home cage with ad libitum access to food and water. Day 1 post‐op rats received Carprofen (5.0 mg/kg; SQ) and moistened chow on the cage floor.

### 4.4 Phenotyping

#### 4.4.1 Timeline

After NG surgery (CCK‐SAP n = 16, SAP n = 21), animals were kept on a chow diet (Figures 1 and 2). After 6 weeks, half of the animals from each group were switched to a HFHS diet for 5 weeks (HFHS n = 8/group; Figure 3) while the rest were maintained on a chow diet (Chow n = 8/group).After 11 weeks of chow, these rats were switched to HFHS diet for 5 weeks (n = 8/group; Figure 4). Throughout the experiment, body weight and food intake were measured twice weekly. CCK satiety tests were performed at 6 weeks post op. IPGTT was performed 3 weeks post‐op in chow fed, and 3 weeks after switching to HFHS diet. A separate cohort of rats received bilateral NG injections of SAP or CCK‐SAP (n = 7/group; Figure 5), had gastric catheter implanted and were maintained on chow diet.

#### 4.4.2 Feeding studies

##### CCK

To validate CCK‐SAP‐induced ablation of CCK receptor expressing NG neurones, we tested the hypophagic responses to CCK in all rats from each cohort. Within subject design was used with each rat receiving counterbalanced vehicle (500 μL; saline) or sulphated CCK octapeptide (CCK‐8S, 4 μg/kg body weight, IP, Bachem, Torrance, CA, USA).^50^ Animals were fasted for 16 hours on wire bottom cages and injected 1 hours into the dark cycle. The injections were administered in a counterbalanced fashion with half animals receiving the drug on first day followed by vehicle injection on second day, and others receiving vehicle on first day followed by the drug injection on second day. Food weight and spillage were manually recorded for 2 hours or in an automated episodic food intake monitoring system (BioDAQ). Rats with greater than 25% reduction in food intake in response to CCK (4 μg/kg; IP) compared to saline were included in the SAP group, and rats with less than 5% satiation after CCK compared to saline were included in CCK‐SAP group. Three rats were excluded based on these parameters.

##### Pre‐load intra‐gastric infusions

Rats were fasted overnight starting at light onset. Beginning at dark onset on the following day, animals received a 10 mL intra‐gastric infusion at 1 mL/minute rate, 30 minutes before refeeding. Infusates were 75% sucrose (Sigma‐Aldrich, MO, USA), 37.5% fat (Microlipid®, Nestle SA, Switzerland) or 2% methyl cellulose (viscosity:15 cP, Sigma‐Aldrich, MO, USA) with three consecutive replicate days and food intake was averaged across the 3 days for each condition.

##### Food intake microstructure

Meal patterns were analysed in a BioDAQ (Research diets Inc, New Brunswick, NJ) episodic food intake monitoring system (n = 8/group). Meals were defined by at least 0.2 g consumed without interruption by a pause of >10 minutes. The system consists of a low spill enlarged opening food hopper placed on an electronic balance mounted together on the animals’ home cage. Chow intake was continuously measured from weeks 3‐6 in chow‐fed rats (Figure 1), or weeks 6‐11 in HFHS (Figure 3).

#### 4.4.3 Energy expenditure

In a separate cohort of chow‐fed rats (n = 6/group), the volume of oxygen consumed (VO2, mL/kg body weight/h) and carbon dioxide produced (VCO2, mL/kg body weight/h) were continuously recorded at 12‐minutes intervals by indirect calorimetry (CLAMS setup; 2‐L/min flow) following a previously published protocol.^111^ The total energy expenditure was computed using the following equation: 3.815 × VO2 (L/h) + 1.232 × VCO2 (L/h), and data were represented as kcal/h. A week before entering CLAMS, rats were provided with powdered chow instead of pellets in a food cup to mimic the CLAMS cage set up. At 6 weeks post‐op, rats were placed in the CLAMS cages. Rats were allowed to acclimatize for 3 days, and indirect calorimetry was performed during days 4 and 5.

#### 4.4.4 Intraperitoneal glucose (IPGTT) tolerance test

After overnight fasting (~16 hours), an IP injection of 50% glucose solution at a dose of 2 g/kg body weight was administered. Glucose concentrations were determined from the tail venous blood using a hand‐held glucometer (Accu‐Chek™ glucose meter, Roche Diagnostics, QC, Canada) at 0, 30, 60 and 120 minutes after glucose injection.

#### 4.4.5 Progressive ratio test

Rats were food restricted to 90% at the starting body weight prior to the behavioural task. They were then conditioned to lick for 20% sucrose or 10% fat solutions (Microlipid®, Nestle SA, Switzerland) in a lickometer box that dispensed <3 µL per lick on a fixed ratio 1 (FR1) schedule for 1 hour per day for 5 days. Once conditioned to the FR1 schedule, rats were trained to lick on a fixed ratio 5 (FR5) schedule for 3 days before they were tested using a progressive ratio breakpoint test.

### 4.5 Statistics

Statistical analysis for the experiments is described in each figure legend and was determined using GraphPad Prism 8.3 software. Two‐tailed unpaired Student's *t* tests were used for comparing two groups; one‐way ANOVA, with or without repeated‐measures, was used for comparing three groups; two‐way ANOVA, with or without repeated‐measures, was used for comparing more than one factor between groups as performed for food intake, energy expenditure, weight gain and blood glucose during IPGTT. Data are presented as mean ± SEM and statistical significance is declared at *P* < .05.

## CONFLICT OF INTEREST

None of the authors declare any conflict of interest.

## AUTHORS’ CONTRIBUTIONS

MM: acquisition of data, analysis and interpretation of data, drafting of the manuscript and statistical analysis. AS, CD, DQ: acquisition of data, analysis and interpretation of data and editing of the manuscript CS: analysis and interpretation of data, editing of the manuscript, obtained funding. GL: study concept and design, acquisition of data, analysis and interpretation of data, drafting of the manuscript, obtained funding and study supervision.
